# Dipole Alignment and Layered Flow Structure in Pressure-Driven Water Transport through MoS_2_ Membranes

**DOI:** 10.1021/acs.jpcb.6c00596

**Published:** 2026-05-30

**Authors:** João Victor Lemos Vale, Lucas Cesena, Bruno H. S. Mendonça, Elizane E. de Moraes

**Affiliations:** † Instituto de Física, 28111 Universidade Federal da Bahia, Campus Universitário de Ondina, Salvador 40210-340, Bahia, Brazil; ‡ Departamento de Física, ICEX, 28114 Universidade Federal de Minas Gerais, CP 702, 30123-970 Belo Horizonte, Minas Gerais, Brazil

## Abstract

Efficient water transport through nanostructure membranes is essential for advancing filtration and desalination technologies. In this study, we investigate the flow of water through molybdenum disulfide (MoS_2_) nanopores of varying diameters using molecular dynamics simulations. The results demonstrate that both pore size and atomic edge composition play crucial roles in regulating water flux, molecular organization, and dipole orientation. Larger pores facilitate the formation of layered water structures and promote edge-accelerated flow, driven by strong electrostatic interactions between water molecules and exposed molybdenum atoms. In narrower pores, confinement and asymmetric edge chemistry induce the ordered alignment of dipoles, thereby enhancing directional transport. Velocity and density maps reveal that pore edges act as active zones, concentrating flow and reducing resistance. Our findings reveal that ordered dipole alignment in subnanometer pores is the fundamental mechanism driving directional water transport. Unlike previous studies focused primarily on desalination, we demonstrate that this molecular orientation is a distinguishing factor that governs flow stability. This discovery suggests that MoS_2_ membranes are not only efficient for filtration but also highly promising for nanofluidic sensors, where controlled molecular orientation is critical for dictating electrical signals and improving device sensitivity.

## Graphical abstract

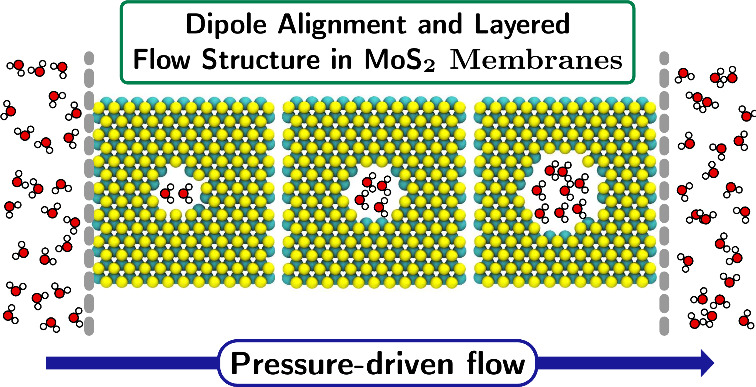

## Introduction

Access to clean water is becoming increasingly difficult in many parts of the world. As climate change intensifies and populations grow, ensuring safe and sustainable water supplies has become one of the most urgent challenges of our time. Recent simulations have highlighted MoS_2_ as a superior material for desalination; for instance, Abal et al.^1^ demonstrated how multilayered structures can effectively filter ions. Additionally, Dillenburg et al.^2^ showed the impact of functionalized nanopores on water flux. However, a critical gap remains in understanding how the intrinsic atomic arrangement of the pore edges, specifically the Mo/S ratio-directly influences the rotational freedom and dipole alignment of water molecules, which we address here.

In response, researchers have turned to innovative technologies like nanofiltration^1,3–10^ and desalination-especially those that use two-dimensional (2D) materials known for their remarkable structural and chemical properties.^11–16^ Graphene was once the star of this field, praised for its strength and thinness. But its chemical stability and the difficulty of creating ultrasmall pores have limited its practical use in large-scale water purification. That is where molybdenum disulfide (MoS_2_)—an inorganic compound of molybdenum (Mo) and sulfur (S) from the transition metal dichalcogenide (TMD) family—comes into the picture. MoS_2_ ^1,17–24^ stands out for its semiconducting nature, mechanical durability, and ability to be chemically modified, making it a strong candidate for next-generation water filtration membranes.^24–36^

MoS_2_ membranes are especially appealing due to the possibility of tailoring their pore structures at the atomic scale, allowing the selective transport of water molecules while rejecting ions and contaminants.^18,37–45^ Such control over molecular transport is critical for desalination, wastewater treatment, and nanofluidic devices. Recent experimental and theoretical studies have shown that MoS_2_ nanopores can outperform conventional polymeric membranes in terms of both water permeability and salt rejection, highlighting their potential for energy-efficient water purification.^18,46,47^

A fundamental step toward realizing these applications is a deep understanding of water flow dynamics through MoS_2_ membranes. Unlike bulk systems, transport at the nanoscale is governed not only by pore size and geometry but also by confinement effects, interfacial interactions, and the specific chemistry of the pore edges.^48,49^ Investigating these aspects experimentally is often limited by constraints on resolution and reproducibility. In this regard, computational simulations provide a powerful complementary approach, enabling atomistic insights into water transport mechanisms, hydrogen-bonding behavior, and the influence of surface functionalization under controlled conditions.^46^

Therefore, the study of water flow in MoS_2_ membranes is not only crucial for optimizing their performance in desalination and filtration but also for advancing fundamental knowledge in nanofluidics. Such understanding can guide the rational design of new materials and devices for critical applications in sustainable water management, energy conversion, and bioengineering.

This study investigates the behavior of water molecules as they flow through MoS_2_ nanopores with varying diameters, utilizing molecular dynamics simulations to capture the intricacies of this process. While previous studies have extensively investigated the role of pore size and edge chemistry, a detailed analysis of dipole alignment and its direct connection to directional transport and edge-enhanced flow remains limited. The findings reveal that both the size of the pore and the chemical nature of its atomic edges are key factors in shaping the dynamics of water transport. MoS_2_ membranes display distinctive flow patterns, marked by accelerated movement near the pore edges and the emergence of layered molecular structures. These phenomena are closely linked to electrostatic interactions between water molecules and the exposed molybdenum and sulfur atoms, which influence how water organizes itself and navigates through the confined space. The remainder of this manuscript is organized as follows: In Sec. II, we present the simulation methodology used in this analysis and define the simulated models. In Sec. III, we discuss the results, and in Sec. IV, we present the conclusions.

## Methodology and Computational Details

To investigate the flux of water molecules through MoS_2_ membranes, we designed the simulation box as illustrated in Figure 1. The cell dimensions are L_*x*_ ≈ 36 Å, L_*y*_ ≈ 37 Å, and L_*z*_ = 70 Å. Periodic boundary conditions were applied in all directions. The system contains two reservoirs filled with 1000 water molecules each, two graphene sheets (piston 1 and piston 2) located at the edges in the z-direction, and a 2D membrane perforated with a nanopore of variable size at its center, located at the origin of the box. We designed membranes with 3 different pore diameters, where the pore diameters are 0.95 nm, 1.22 nm, and 1.63 nm. These values were obtained from the pore diameters of carbon nanotubes (CNT) (7,7), (9,9), and (12,12) (see Figure 2).

**Figure 1:**
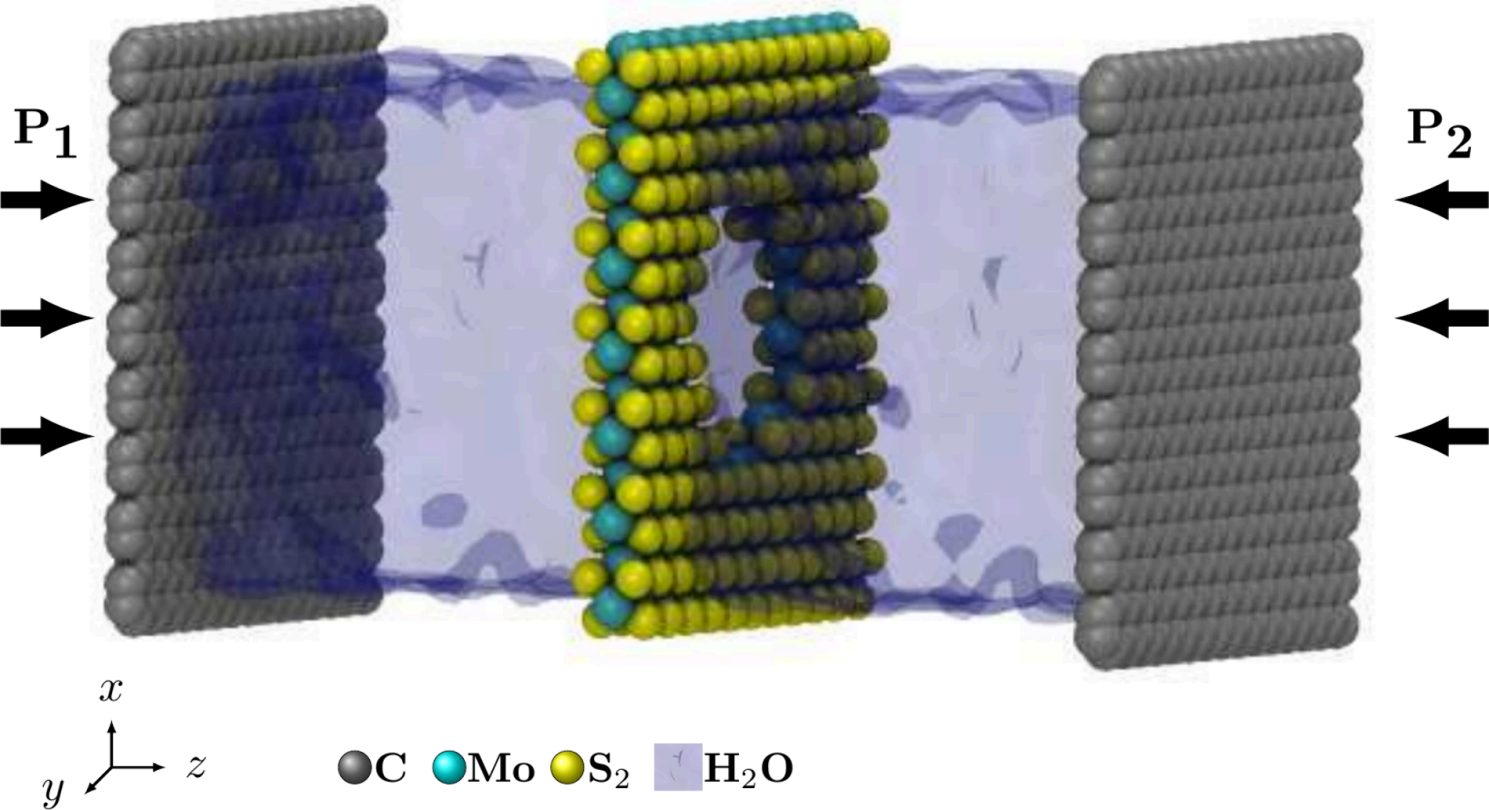
Side view of the molecular dynamics simulation box. The system consists of two water reservoirs (1000 molecules each) separated by a single-layer MoS_2_ membrane with a central nanopore. Pressure-driven flow is induced by applying external forces (*F*) on graphene pistons (gray sheets) to maintain a pressure difference Δ*P* = *P*_1_ – *P*_2_. The coordinate system shows the flow along the *z*-axis, with the *y*-axis oriented out-of-plane.

**Figure 2:**
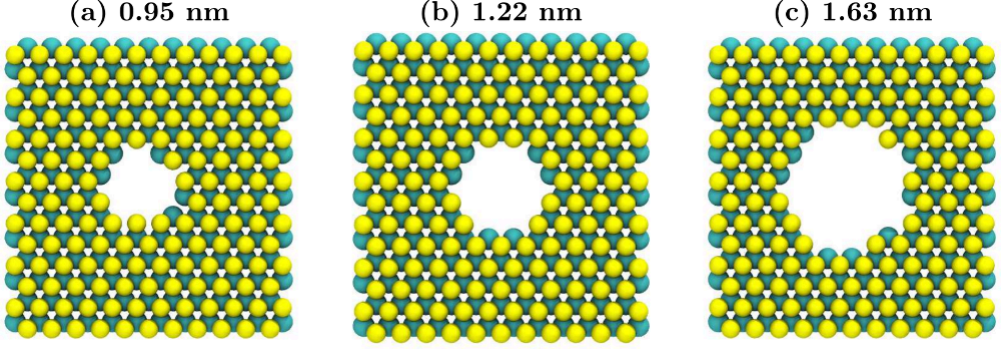
Geometric characterization of the simulated MoS_2_ nanopores. The panels display the three pore sizes investigated: (a) 0.95 nm, (b) 1.22 nm, and (c) 1.63 nm. The pores were designed by removing atoms from the MoS_2_. The structures highlight the exposed Mo (cyan) and S (yellow) atoms at the edges, which regulate water transport.

The membranes and pistons are treated with rigid bond lengths and angles, modeled by the Lennard-Jones potential (LJ) and, for the case of MoS_2_, the Coulombic potential also. To ensure a high-fidelity representation of the fluid properties, we employed the TIP4P/2005 water model.^50^ This choice is motivated by its superior performance in reproducing the phase diagram, density-temperature behavior, and structural organization of water under nanoconfinement compared to standard three-site models. Regarding the influence of polarization, while explicit electronic polarizability could lead to localized increases in interaction strength, the TIP4P/2005 model implicitly accounts for part of these effects through its optimized parametrization of the fixed-charge distribution. Given that the reported dipole alignment and edge-driven flow are primarily governed by the broken symmetry of the MoS_2_ lattice, our results represent a robust description of the atomistic transport mechanisms. In this water model, there is only one LJ interaction site placed at the oxygen position with parameters σ_*OO*_ and ϵ_*OO*_. The positive charges *q*_*H*_ are placed at the hydrogen positions, while the negative charge *q*_*M*_ is placed at a virtual massless site M located at the H-O-H bisector, *d*_*OM*_ distance units apart from the oxygen atom. The cross-interaction parameters between all the species are provided by Lorentz-Beherlort mixing rules. All the force field parameters are summarized in Table 1.

**Table 1:** Force Field Parameters Utilized^a^

H_2_O^a^	MoS_2_ ^b^	Gr^c^ (Pistons)
ϵ_*OO*_ = 0.1852	ϵ_*Mo*_ = 0.0135	ϵ_*CC*_ = 0.086
ϵ_*HH*_ = 0.0000	ϵ_*SS*_ = 0.4612	σ_*CC*_ = 3.40
σ_*OO*_ = 3.1589	σ_*Mo*_ = 4.200	*q*_*C*_ = 0.00
σ_*HH*_ = 0.0000	σ_*SS*_ = 3.130	
*q*_*M*_ = −1.1128	*q*_*Mo*_ = 0.6	
*q*_*H*_ = 0.5564	*q*_*S*_ = −0.3	
*r*_*OH*_ (Å) = 0.9572		
θ_*HOH*_ (deg) = 104.52		
*d*_*OM*_ (Å) = 0.1546		

a The units of the LJ parameters are kcal.mol^–1^ for *ϵ*_*XX*_ and Å for *σ*_*XX*_, where *X* = (C, O, H, Mo, S). The charges are given in elementary charge (*e*) units.

b Vega and Abascal.^50^

c Liang et al.^51^

d Hummer et al.^52^

The simulations were performed by using the Large-scale Atomic/Molecular Massively Parallel Simulator (LAMMPS) software, with a time step of 1.0 fs. The SHAKE algorithm was employed to maintain the bond lengths and angles constrained. Coulombic long-range interactions were computed by the PPPM solver with a precision of 10^–4^. We used a cutoff of 12 Å for both LJ and Coulombic interactions. The simulation protocol is given as follows:

1. Pre-equilibration in the NVE ensemble via a 0.1 ns molecular dynamics run. During this stage, the pistons remain frozen (acting as fixed rigid walls); although the fluid exerts forces on the pistons, these forces are not integrated, ensuring that the piston atoms undergo no displacement.
2. Equilibration in the NVT ensemble at 300 K during 0.2 ns.
3. Forces are then applied in the pistons to impose 1 bar to reach the water equilibrated densities at 300 K using the NPT ensemble for a 1.0 ns MD run.
4. Pistons are frozen in the new equilibrium position, and another NVT equilibration at 300 K is performed for 10 ns.
5. Nanopores are opened by removing the buffer atoms to enable water transport through the pore, and different forces are applied in each piston to mimic the pressure differences using the NPT ensemble during 10 ns at 300 K and different feed pressures.

The water flows along the *z*-axis, normal to the membrane, driven by the pressure difference between both sides of the membrane. The pressure difference is caused by pistons 1 and 2, as illustrated in Figure 1, being introduced by applying external forces F on each atom of the pistons in the z-direction

1 $$F=\frac{P\cdot A}{n}$$

where *n* is the number of atoms, *A* is the surface area, and *P* is the pressure on the surface. Piston 2 (right) has a fixed pressure of 0.1 MPa, while piston 1 (left) was set to achieve left-right pressure differences of 100 MPa, 200 MPa, 300 MPa, 400 MPa, and 500 MPa.

We study the dynamics of the liquid, considering the flow rate of water molecules through the nanopore

2 $$\phi_{H_{2}O}=\lambda V_{mol}v$$

where λ (molecules μm^–1^) is the linear number density of water molecules, *V*_*mol*_ (μm^3^ molecules^–1^) is the average volume of a water molecule and *v* (μm s^–1^) is the water flow velocity which is acquired from the least-squares linear regression line fitted to the data cloud which relates the average molecular displacement as a function of the time, as taken from the MD trajectory file. The calculations are performed inside a rectangular box centered at the pore, and with dimensions spanning the whole supercell in surface directions, and Δ*z* = 4Å along the direction of the flow.

The axial velocity maps were obtained from the same region used in the flow calculations. This region was further partitioned into smaller cells with dimensions Δx=Δy=0.2Å and Δz=4Å . For each cell, the particle average of axial velocity was calculated for a given time *t*, as described by eq 3.

3 $$\overset{®}{v\left(t\right)}=\frac{1}{N_{O}}\overset{N_{O}}{\underset{i}{\sum }}\left|v_{i}\left(t\right)\right|$$

Here, *N*_*O*_ is the number of oxygen atoms within the cell, which are taken as an approximation of the center of mass, and *v*_*i*_(*t*) is the axial velocity of particle *i*. To reduce noise, $\overset{®}{v\left(t\right)}$ was time-averaged over the interval during which the water flow is maintained.

## Results and Discussion

Molecular dynamics simulations reveal that water flows through molybdenum disulfide (MoS_2_) membranes significantly faster than graphene membranes of comparable pore diameters.^53^ The water flux observed in Figure 3 demonstrates the influence of pore diameter and applied pressure on this transport. The increase in pore diameter in MoS_2_ membranes is directly correlated with a significant increase in water flux. This phenomenon can be explained by the reduction in hydrodynamic resistance and changes in the behavior of water molecules within the nanopore. A larger pore allows a greater number of water molecules to position themselves at the channel opening in a less confined manner. With the reduced energy barrier, water molecules adjacent to the pore face and within it experience an acceleration in transport velocity (transmembrane velocity) in the direction of the applied pressure, resulting in significantly higher flux. This velocity phenomenon and layering within the pores are illustrated in the annualized density and velocity maps.

**Figure 3:**
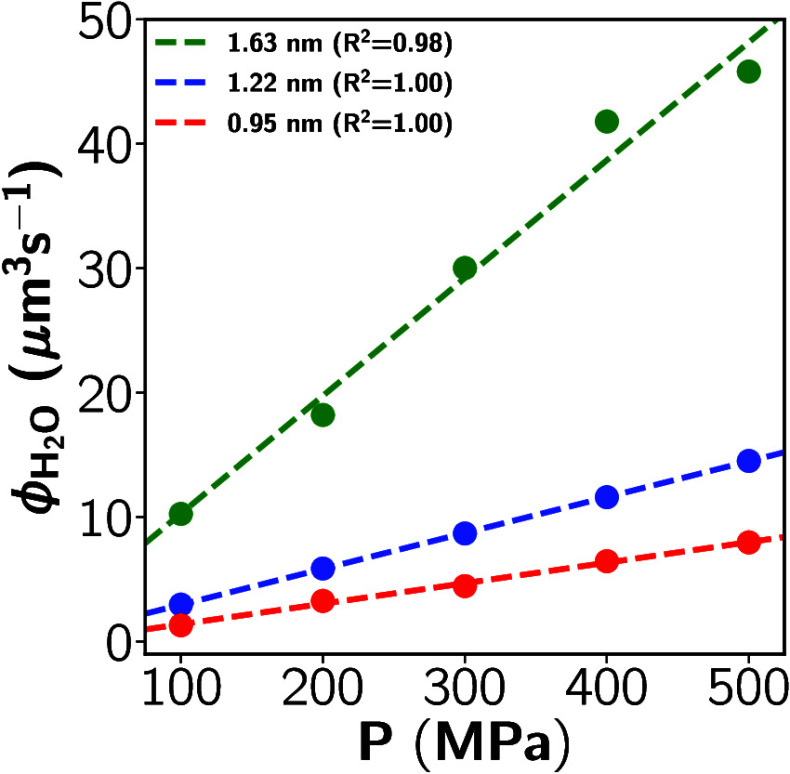
Water flow rates $\left(\phi_{H_{2}O}\right)$ as a function of applied pressure (Δ*P*) for MoS_2_ nanopores. The dashed lines represent the linear regression fits for pore diameters of 0.95 nm (red), 1.22 nm (blue), and 1.63 nm (green). The high *R* ^2^ values (0.98 to 1.00) indicate that the system operates within the linear-response regime for the pressure range of 100 to 500 MPa.

As can be seen in Figure 4, which shows the density maps of water molecules within each MoS_2_ pore studied, increasing pore diameter also influences how water molecules organize and move through the channel. In very narrow pores, such as those with a diameter of 0.92 nm, water molecules can be forced to pass in a single file or in highly ordered hydrogen-bonded chains. Although this confinement can, in some cases, generate a high velocity for the confined chain due to the high pressure concentrated in the small orifice, the accommodation capacity is limited. With increasing pore diameter (e.g., for diameters of 1.22 and 1.63 nm), the transport channel can accommodate more water molecules, and consequently, this leads to an increase in molecular density, which ultimately dominates the effect and results in a net increase in water flux.^18,54,55^ An important factor observed in all pores and clearly demonstrated in the density maps is the influence of the atomic termination at the pore edge, which plays a crucial role and is the main mechanism that modulates water flow. The preferential attraction of water molecules to Mo atoms compared to S atoms, and its effect on flow, can be explained by the intrinsic polarity and hydrophilicity/hydrophobicity of these sites. The explanation lies in the fact that in molybdenum disulfide, Mo is less electronegative than S. Consequently, the Mo atoms carry a partial positive charge. The exposure of these Mo sites at the nanopore edge creates highly hydrophilic sites, meaning they attract more water molecules.^53,56^ As is known, water is a polar molecule with a significant dipole moment. The oxygen atom carries a partial negative charge, while the hydrogen atoms carry partial positive charges. The oxygen in the water molecule is strongly attracted to the Mo sites at the pore edge, resulting in a favorable and stronger electrostatic interaction compared to the S sites. The S atoms are more electronegative and carry a partial negative charge. When exposed at the pore edge, these sites are less likely to interact with the O in the water and can, in some configurations, act as more hydrophobic (water-repellent) surfaces compared to the Mo border. The electrostatic repulsion between similar partial negative charges hinders the entry and organization of water molecules. Although these factors influence water flow through MoS_2_ pores, in general, what we can observe is that the mobility of water molecules flowing in the pores is highly governed by the pore size and the applied pressure gradient, leaving the interaction between the attraction or repulsion of water molecules with the MoS_2_ atoms in the pores to secondary importance.^53,56^

**Figure 4:**
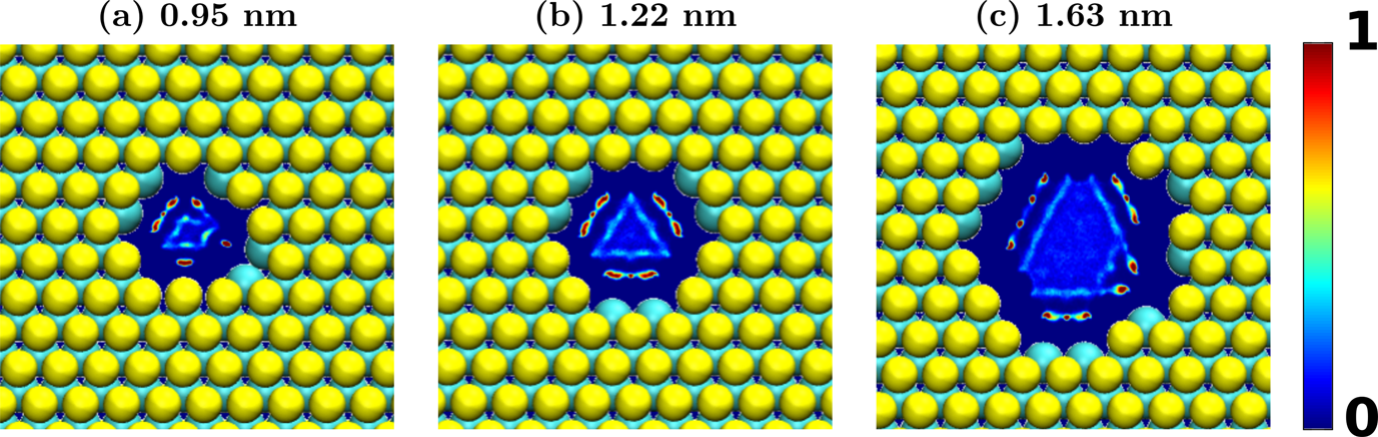
Oxygen density maps within the MoS_2_ nanopores. The maps were obtained under a pressure difference of Δ*P* = 100 MPa for pore diameters: (a) 0.95 nm, (b) 1.22 nm, and (c) 1.63 nm. Red regions indicate high water occupancy, revealing a clear layered structure and a preference for molecules to reside near the molybdenum-rich edges. The blue regions indicate low probability.

The distribution of dipole angles along the x-direction for pores of 0.95 nm, 1.22 nm, and 1.63 nm under a pressure gradient of Δ*P* = 100 MPa is shown in Figure 5. For the 1.22 and 1.63 nm pores, dipole orientations appear broadly dispersed and randomly distributed, reflecting the greater geometric symmetry and spatial freedom within the channel. This freedom allows water molecules to adopt multiple orientations without a dominant directional preference. In contrast, the 0.95 nm pore imposes significant confinement, which restricts molecular rotation and induces a more ordered dipole alignment. This alignment is driven by both spatial constraints and asymmetric surface interactions at the pore edge. The asymmetry is from the atomic structure of MoS_2_, where Mo and S atoms are distributed unevenly along the pore edge. In many cases, the pore opening exposes predominantly Mo atoms on one side and S atoms on the opposite side, generating an asymmetric electrostatic field along the pore axis. Mo atoms, carrying partial positive charges, strongly attract the negatively charged oxygen atoms of water. Conversely, S atoms, being more electronegative and also partially negative, tend to repel oxygen and interact weakly with the hydrogen atoms. This chemical and charge distribution at the pore edge leads to a preferential dipole orientation: oxygen atoms point toward Mo sites, while hydrogen atoms orient toward S atoms. The result is a directional dipole alignment along the pore axis, which is clearly visible in the density maps (Figure 4) and is consistent with the observed flow behavior. This pattern is especially pronounced in the 0.95 nm pore, where confinement amplifies edge interactions and limits rotational freedom, promoting highly ordered molecular organization.

**Figure 5:**
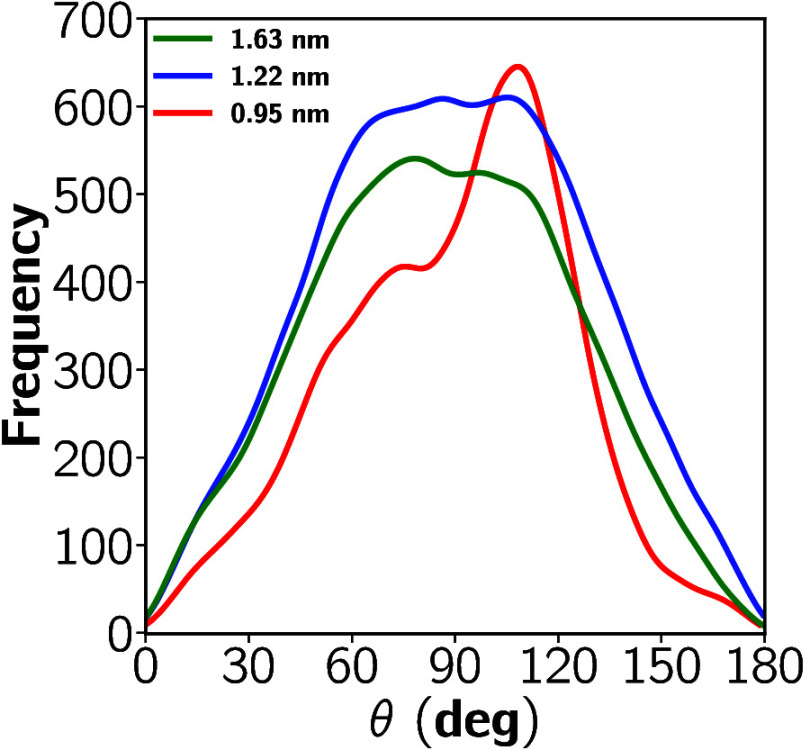
Angular distribution of water dipoles (θ) inside MoS_2_ nanopores. The probability distribution is shown for diameters of 0.95 nm (red), 1.22 nm (blue), and 1.63 nm (green) at Δ*P* = 100 MPa.

To understand the mechanisms responsible for the water flux shown in Figure 3, we evaluated the axial velocity profiles of water molecules passing through the membrane an applied pressure difference of 100 MPa, as shown in Figures 6(a)–(c). The axial velocity profiles of water were computed by selecting a region of interest in the shape of a parallelepiped with dimensions (d*x* = *L*_*x*_), (d*y* = *L*_*y*_), and (d*z* = 4) Å, where the thickness along the *z* direction is the same as that adopted in the flux calculation. This region was then subdivided into smaller bins with dimensions (d*x* = *L*_*x*_/*N*), (d*y* = *L*_*y*_/*N*), and (d*z* = 4) Å, where N is the number of partitions along each in-plane direction. For each bin and at each time step, the magnitude of the z-component of the velocity, |*v*_*z*_|, was calculated for every oxygen atom within that bin, taking the oxygen atom position as an approximation to the molecular center of mass of the water molecule. These values were then averaged over all oxygen atoms present in the bin at that time step. This procedure was repeated for all time steps considered in the analysis. Finally, a time average of |*v*_*z*_| was computed for each bin, so that the resulting value already corresponds to an average both over the oxygen atoms within the bin and over the trajectory frames. The final velocity map is then constructed from an output file containing the (x, y) coordinates of each bin, along with the corresponding mean value of |*v*_*z*_|. The velocity was converted to m/s in order to follow the convention adopted in the literature^21,57,58^

**Figure 6:**
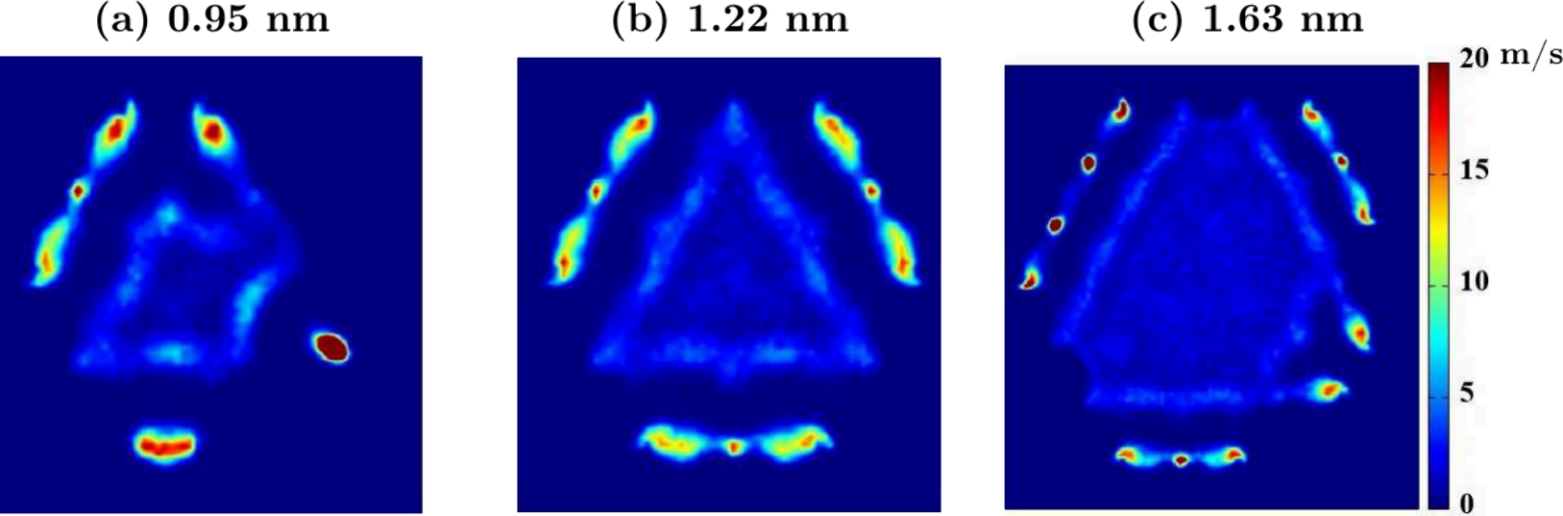
Axial velocity profiles of water molecules across the MoS_2_ nanopores. The time-averaged velocity in the z-direction is shown for pore diameters of (a) 0.95 nm, (b) 1.22 nm, and (c) 1.63 nm under Δ*P* = 100 MPa. Red regions indicate high water occupancy, revealing a clear layered structure and a preference for molecules to reside near the molybdenum-rich edges. The blue regions indicate low probability.

The analysis of axial velocity profiles of water molecules, Figure 6, shows that, regardless of pore diameter, the highest velocities are consistently located near the edges of the channel. This behavior contrasts with classical laminar flow, where maximum velocity typically occurs at the center. This deviation from classical continuum behavior highlights the importance of atomistic interactions in nanoscale transport. In MoS_2_ nanopores, this inversion is attributed to strong electrostatic interactions between water molecules and the exposed molybdenum atoms at the pore edge, which act as highly hydrophilic sites. As shown in the density maps (Figure 4), there is a significant accumulation of water molecules near the pore edges, forming structured layers that promote coordinated flow. Additionally, the preferential dipole alignment observed in narrow pores (Figure 5) contributes to the stability of molecular trajectories, reducing fluctuations and enabling high velocities along the edges. This peripheral flow pattern is a distinctive feature of water transport in MoS_2_ and may be leveraged to enhance membrane performance in filtration and molecular separation applications. Directly, the speed of water molecules is higher in Mo-rich regions because these areas create a low-friction (high hydrodynamic) flow surface. This behavior can be interpreted in terms of reduced effective interfacial resistance at Mo-rich regions, where favorable electrostatic interactions and local molecular ordering promote preferential transport pathways and enhanced axial velocities. This interpretation is consistent with previous studies of nanoconfined water transport. In particular, Aluru et al.^58^ have shown that enhanced flow in carbon nanotubes and graphene nanopores is associated with reduced interfacial friction and partial slip at the solid–liquid interface, which significantly increases water velocity compared to classical continuum predictions. In our case, although the microscopic origin differs due to the presence of heterogeneous Mo and S edge terminations, the emergence of preferential high-velocity regions near the pore edges suggests a similar reduction in interfacial resistance governing the transport. In addition, this edge-enhanced velocity profile represents a significant departure from the behavior observed in graphene or even in multilayered TMD systems. Unlike the findings in Abal et al.,^1^ where interlayer friction plays a dominant role, our single-layer results reveal that electrostatic attraction at Mo sites creates a coordinated flow layer that minimizes traditional hydrodynamic resistance.

The accumulated count of water molecules over time, Figure 7, offers a clear and intuitive way to understand how efficiently each pore transports water. What we see is straightforward: the larger the pore, the more water gets through – and faster. The 1.63 nm pore leads the way, followed by the 1.22 nm and then the narrower 0.95 nm channel. This is not just about size; it is about how water molecules arrange themselves inside the channel. As shown in the density maps (Figure 4), wider pores give water molecules room to organize into layered structures, especially near the edges – the same regions where velocity peaks (Figure 6). This layered flow, combined with edge acceleration, enhances transport efficiency. In narrower pores, dipole alignment (Figure 5) helps stabilize the flow, but the tight geometry limits how many molecules can pass through at once. Therefore, the water molecule count reflects the interplay between pore geometry, transport dynamics, and molecular organization, reinforcing pore diameter and symmetry as the dominant factor in flow efficiency.

**Figure 7:**
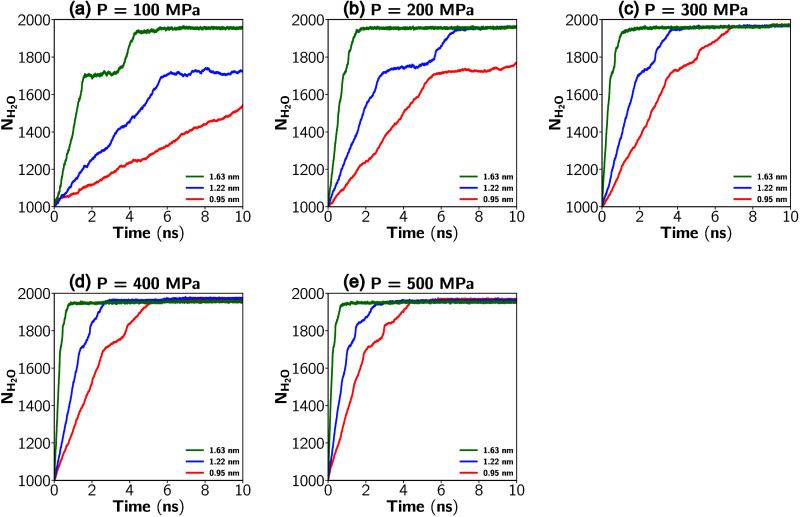
Cumulative number of water molecules $\left(N_{H_{2}O}\right)$ permeated through MoS_2_ nanopores as a function of simulation time. Panels (a–e) represent applied pressure differences from 100 to 500 MPa. In each case, the transport rates for diameters of 0.95 nm (red), 1.22 nm (blue), and 1.63 nm (green) are compared.

Although the applied pressure differences in this work are higher than those typically used in experimental desalination processes, this approach is commonly adopted in molecular dynamics simulations to obtain measurable transport within accessible timescales. At experimentally relevant pressures (e.g., 1–5 bar), the characteristic flow occurs over much longer timescales, making direct simulation computationally prohibitive.

Importantly, the water flux shows approximately linear dependence on the applied pressure difference (see Figure 3), indicating that the system operates close to the linear-response regime over the range of pressures considered. Therefore, the observed transport mechanisms are expected to remain valid under experimentally relevant conditions.

In addition, to facilitate comparison with experimental data, we have calculated the water permeability, providing a more direct connection between the simulation results and measurable macroscopic quantities. The hydraulic permeability - commonly referred to as permeability *L*_*p*_ is a parameter that combines properties of both the membrane and the fluid. Phenomenologically, it relates the volumetric flow rate, ϕ_*water*_, to the pressure drop, Δ*P*, across the membrane, as expressed by eq 4 ^59^

4 $$\frac{\phi_{water}}{A}=L_{p}\Delta P$$

Here, *A* is the total membrane area, calculated as *A* = *L*_*x*_ *L*_*y*_. Therefore, we obtained *L*_*p*_ from the slope of the regression line fitted to the plot of $\frac{\phi_{water}}{A}$ as a function of Δ*P*. The calculated values are 460.22, 804.52 and 2639.97 for pore sizes of 0.95 nm, 1.22 nm and 1.63 nm respectively. These values are expressed in units of L/m^2^.h.bar to allow comparison with previous results from literature.^3,9,57,60–62^

## Conclusions

In this work, we show that dipole alignment and edge-driven flow act as complementary mechanisms governing transport in MoS_2_ nanopores, providing a more complete microscopic picture beyond conventional geometric and hydrodynamic descriptions. While previous literature emphasized the “anchor mechanism” and interlayer friction as primary regulators of flux in thick membranes, our results isolate the ordered dipole alignment as the critical differentiator in single-layer systems. We demonstrate that the intrinsic atomic asymmetry of the MoS_2_ lattice does not merely hinder or facilitate flow, but actively organizes it into structured molecular layers. This discovery shifts the paradigm of MoS_2_ membranes from simple passive filters to active nanofluidic components. The high sensitivity of water orientation to the pore’s chemical environment suggest that these structures are ideally suited for next-generation nanofluidic sensors. In such devices, the predictable alignment of dipoles can be directly correlated to specific electrical signals, offering a robust platform for environmental monitoring and early disease diagnosis through molecular-scale detection. Water flows through MoS_2_ nanopores much more efficiently than through graphene pores of similar size. As the pore diameter increases, so does the water flux—not just because there’s more space, but also because the molecules themselves behave differently. In wider pores, water molecules can spread out, organize into layers, and move with less resistance, especially near the edges of the channel. The density and velocity maps indicate that these edges are not just boundaries, but active zones. Water tends to accumulate and accelerate along the pore edges, where molybdenum atoms create highly attractive, hydrophilic sites. This edge-driven flow diverges from the classic concept of laminar movement, reminding us that at the nanoscale, atomic details are crucial. The dipole orientation results reinforce this: in narrower pores, water molecules align their dipoles in response to confinement and the asymmetric distribution of Mo and S atoms, creating a directional flow that is both ordered and efficient. Altogether, these findings highlight how pore geometry, edge chemistry, and molecular orientation collectively shape water transport in MoS_2_ membranes. Understanding these interactions helps explain why these systems are so permeable and opens the door to designing smarter, more effective nanofluidic and filtration technologies using transition metal dichalcogenides.
